# Sinonasal inverted papilloma: rate of recurrence and malignant transformation in 44 operated patients

**DOI:** 10.1016/j.bjorl.2019.07.003

**Published:** 2019-08-12

**Authors:** Mariana Ferreira Sbrana, Renata Ferraz Rafael Borges, Fábio de Rezende Pinna, Deusdedit Brandão Neto, Richard Louis Voegels

**Affiliations:** Universidade de São Paulo (USP), Departamento de Otorrinolaringologia, São Paulo, SP, Brazil

**Keywords:** Papilloma, Paranasal sinuses, Benign neoplasms, Malignancy, Recurrence

## Abstract

**Introduction:**

Although sinonasal inverted papillomas are benign lesions, they are locally aggressive and have a potential malignant transformation ranging from 5% to 15%, with a high recurrence rate.

**Objective:**

The aim of this article is to describe the rate of recurrence and malignant transformation in patients with a diagnosis of inverted papilloma who underwent surgery in a tertiary hospital in São Paulo.

**Methods:**

We performed a retrospective analysis of patients diagnosed with sinonasal papilloma who had undergone surgery in a tertiary hospital in São Paulo, between August 1998 and August 2017. A patient chart review was conducted to assess data of patients’ demographics, tumors characteristics, follow-up appointments, recurrence and malignancy. Inverted papillomas were analyzed and classified under the Krouse staging system.

**Results:**

A total of 69 surgeries were performed in patients with diagnosis of sinonasal papilloma. Inverted papilloma was the most prevalent subtype (49 cases ‒ 80.33%), followed by exophytic papilloma (6 cases ‒ 9.84%) and by oncocytic papilloma (6 cases – 9.84%). The recurrence rate was 34.09% for inverted papilloma (15/44) and the mean time of recurrence was 24.6 months. Malignant transformation occurred in 6 patients (13.64%). Three of these patients presented carcinoma in the first surgery and three patients developed carcinoma during the follow-up.

**Conclusion:**

The high recurrence rate and malignancy potential allow us to consider inverted papillomas as aggressive tumors. In a tertiary hospital in São Paulo the recurrence rate the mean time to recurrence is 24.6 months. The recurrence after 10 years implies was 34.09% and the need for long-term follow up. It is possible that the high recurrence rate and the high malignant transformation rate we found are due to the large number of tumors discovered at an advanced stage (most of them staged T3 and T4), secondary to poor access to health system, in developing countries.

## Introduction

Sinonasal papillomas are benign tumors that originate from Schneiderian mucosa that lines both nasal cavity and paranasal sinuses. According to the World Health Organization papillomas are classified in three different subtypes: exophytic (squamous), inverted, or oncocytic (or cylindrical cells).^1^ They are relatively rare, with an incidence of 0.75–1.5/100,000 per year and the inverted papilloma is the most prevalent subtype.^2^

Although Sinonasal Inverted Papillomas (SNIP) are benign lesions, they are locally aggressive and have a potential for malignant transformation ranging from 5% to 15%, with a high recurrence rate (up to 78%).^3^ The aim of this article is to describe rate of recurrence and malignant transformation in patients with diagnosis of SNIP who underwent surgery in a tertiary hospital in São Paulo.

## Methods

We performed a retrospective analysis of patients diagnosed with sinonasal papilloma who had undergone surgery in a tertiary hospital in São Paulo, between August 1998 and August 2017. This study was submitted and approved by the hospital ethical committee (nº 2.395.820). A patient chart review was conducted to assess data such as age, sex, medical history, alcohol and tobacco exposure, number of previous sinus surgeries, type of surgery performed, location of the tumor, histopathological subtype, staging, follow-up appointments, recurrence and malignancy.

According to the findings in nasal and paranasal Computed Tomography (CT) imaging, Magnetic Resonance Imaging (MRI) and intraoperative findings (Table 1) inverted papillomas were then analyzed and classified under Krouse staging system.^4^ Authors analyzed the CT images and, if the tumor could not be differentiated from inspissated secretions, MRI images were then analyzed. Intraoperative findings were reviewed to confirm the extent of the papilloma.

**Table 1:** Krouse staging system.^4^

T	Tumor location
T1	Tumor confined to the nasal cavity, without extension into the sinuses.
T2	Tumor involving the osteomeatal complex and ethmoid sinuses, and/or the medial portion of the maxillary sinus, with or without involvement of nasal cavity.
T3	Tumor involving the lateral, anterior, posterior, inferior or superior walls of maxillary sinus, the sphenoid sinus, and/or the frontal sinus, with or without criteria to T2.
T4	Tumor with extranasal/extrasinus extension, to involve adjacent, contiguous structures, such as the orbit, the intracranial compartment or the pterygomaxillary space or tumors associated with malignancy.

After surgery, patients were evaluated once a week for one month, then again at 3 months, at 6 months, at one year post-op and then annually. In each visit patients were asked about their symptoms and an endoscopic exam was performed to assess the recurrence of papilloma. Patients with recurrent nasal symptoms (nasal blockage, rhinorrhea, headache, epistaxis) or with suspicious lesions identified in endoscopic exam were submitted to a new CT scan and biopsy.

## Results

A total of 69 surgeries were performed in patients with diagnosis of sinonasal papilloma between August 1998 and August 2017. Eight patients were excluded because of lack of data in medical records, resulting in a total of 61 patients. Inverted papilloma was the most prevalent subtype, corresponding to 49 cases (80.33%), followed by exophytic papilloma (6 cases – 9.84%) and by oncocytic papilloma (6 cases – 9.84%). Three patients with inverted papilloma subtype were diagnosed with Squamous Cell Carcinoma (SCC) at the time of first surgery at our hospital. Table 2 summarizes demographics in each subtype of papilloma.

**Table 2:** Demographics by papilloma subtype.

	Inverted	Exophytic	Oncocytic
Mean age	57.18	46.5	64.33
Sex (Female, %)	40.81%	50%	66.67%
Prior sinus surgery	14.28%	16.67%	0
Smoking	18.37%	33.33%	33.33%
Alcoholism	6.12%	16.67%	0

Endoscopic sinus surgery alone was performed in 43 patients (70.49%), combined endoscopic and external approach (Calldwel-Luc or lateral rhinotomy or Lynch) were performed in 15 patients (24.59%) and external approach alone was performed in 3 patients (4.92%). There was no major complication in any case.

Krouse classification was applied to all SNIP. One patient (2.04%) was classified as Stage T1, 14 patients as Stage T2 (28.57%), 29 patients as Stage T3 (59.18%) and five patients as Stage T4 (10.20%). The majority of SNIP originated from ethmoid sinuses (56.52%), followed by maxillary sinus (45.65%), lateral nasal wall (17.39%), papyracea (13.04%), frontal recess (6.52%), skull base (6.52%), inferior turbinate (4.35%), sphenoid (2.17%) and nasal septum (2.17%).

Four patients were lost follow-up and one patient was referred to an oncologic service to undergo chemotherapy and radiotherapy because the anatomopathologic result showed SCC and the tumor could not be completely removed at the time of surgery. Considering the 44 patients that continued to be treated, the mean follow-up for SNIP was 35.56 months.

Recurrence rate was 34.09% for inverted papilloma (15/44) and mean time of recurrence was 24.6 months (ranging from 1 to 128 months). Of the recurrent inverted papillomas, 12 were at Stage T3 (80%), two at Stage T4 (13.34%) and one at Stage T2 (6.67%). Majority of recurrent lesions had multiple insertions. Ethmoid was the site of origin in 73.34% of recurrent cases, followed by maxillary in 66.67% of recurrent papillomas. Table 3 demonstrates the site of origin of primary and recurrent lesions.

**Table 3:** Location of primary and recurrent tumor.

Tumor location	Primary location	Recurrence location
Ethmoid	26 (56.52%)	11 (73.34%)
Maxillary	21 (45.65%)	10 (66.67%)
Lateral nasal wall	8 (17.39%)	4 (26.67%)
Papiracea	6 (13.04%)	2 (13.34%)
Frontal recess	3 (6.52%)	2 (13.34%)
Skull base	3 (6.52%)	1 (6.67%)
Inferior turbinate	2 (4.35%)	0
Sphenoid	1 (2.17%)	0
Nasal septum	1 (2.17%)	0

Malignant transformation occurred in 6 patients (13.64%). Three of these patients presented SCC in the first surgery (synchronous malignancy) and three patients developed carcinoma during the follow-up at 11, 48 and 54 months after first surgery in our service (metachronous malignancy). Five of these patients were female (83.34%), five were classified as Stage T4 (83.34%) and one patient was classified as Stage T3 (16.67%). Four patients with malignant lesions had already undergone previous sinus surgery (66.67%) (Table 4).

**Table 4:** Demographics of population with and without malignant transformation.

	Inverted papilloma with malignant transformation	Inverted papilloma without malignant transformation
Mean age	59.84	58.34
Sex (Female, %)	83.34%	35.9%
Previous sinus surgery	66.67%	12.82%
Tobacco exposure	16.67%	20.51%
Alcoholism	16.67%	5.13%
T1	0	2.56%
T2	0	28.2%
T3	16.67%	69.23%
T4	83.34%	0

## Discussion

Inverted papilloma is the most common diagnosed subtype of sinonasal papilloma, accounting for more than 50% of cases and followed by exophytic subtype, with a similar prevalence. Oncocytic papilloma is rare, comprising 3–5% of cases.^5^ In this study, a higher prevalence of inverted papilloma was found, comprising 80.33% of all papillomas. Exophytic subtype was found in only 9.84% of sinonasal papilloma as well as oncocytic subtype.

The mean age for SNIP patients was 57.18 years and a ratio male/female of 1.45:1 was found in our study. This was consistent with current literature that states SNIP mainly affects patients in their fifth decade of life and has a preponderance in males.^6^, ^7^ Exophytic papilloma often occurs in younger patients, in about their forties^5^ and oncocytic papilloma usually occurs in older patients.^8^ Our findings corroborate these data, with a mean age of 46.5 years for exophytic subtype and a mean age of 64.33 years for the oncocytic papilloma.

Ethmoid sinus was the major site of origin of SNIP, followed by maxillary sinus. These finding differ from current literature in which maxillary sinus has been described as inverted papilloma’s most common site of origin.^9^, ^10^, ^11^

Pathogenesis of SNIP is not completely known. Some theories associate them with infection by Human Papilloma Virus (HPV). A 2013 meta-analysis by Syrianen and Syrianen found a prevalence of 38.5% of HPV infection in patients with sinonasal papilloma and a 37.8% HPV-positivity in inverted papillomas with no statistically significant difference based on the HPV method detection.^12^ A 2014 review by Govindaraj and Wang demonstrated association between HPV infection and inverted papilloma and suggested the virus plays a role in the pathogenesis of the tumor and in its progression to dysplasia and squamous cell carcinoma.^6^ Zhao et al. found a statistically significant association between HPV infection and malignant transformation in inverted papillomas.^13^ In contrast, Mohajeri et al., in a recent study, analyzed 76 patients with SNIP and detected a 13.2% HPV positivity, using PCR method, suggesting that HPV is an unlikely etiology of tumor development. Furthermore, they had found absence of HPV-positivity among SCC patients, which suggests the virus is not a contributing factor for malignant transformation.^14^

SNIPs are associated with malignancy, with a malignant transformation rate of 1.9–15%.^5^, ^7^, ^15^, ^16^, ^17^ SCC is the predominant histologic type developed, but mucoepidermoid carcinoma and sinonasal undifferentiated carcinoma have also been described.^5^, ^16^ A malignant transformation rate of 13.64% was found in our study (all cases with SCC). All these patients were staged at T3 or T4 in Krouse staging system, which suggests the extension of the tumor might have an association with malignant transformation.

Malignancy should be considered when there is a rapid tumor growth, invasion of adjacent structures or nasal bleeding.^7^ There is no consensus about the role of HPV infection in malignant papilloma. Some authors have found a similar detected rate of HPV infection in SCC ex-papilloma and Schneiderian papilloma without malignancy.^16^, ^17^, ^18^, ^19^, ^20^ However, there are some studies which suggest HPV-16 and HPV-18 infection can play a role- inducing malignancy once there is a high detection rate of these subtypes in inverted papillomas with moderate to severe dysplasia or SCC. Moreover, a 2016 meta-analysis suggested a statistically significant association between high-risk HPV (especially HPV-18 infection) and malignant papilloma.^13^

Genetic alterations concerning proapoptotic factors such as p53, p21, p27, p16, inflammatory genes (COX-2), antiapoptotic factors (p63, bcl-2) and intercellular adhesion molecules (desmoglein 3, e-cadherin, catenin, fascin) may play a role into SNIP malignant transformation.^17^ Recently, Undager et al. identified identical EGFR (epidermal growth factor receptor) genotypes in matched pairs of SNIP and SNIP-associated SCC, providing genetic evidence of a link between these tumors and suggesting a role of activating EGFR mutations in the pathogenesis of SNIP and SNIP-associated SCC.^21^

Median survival time of patients with SCC ex-papilloma is about 62.2 months and the 5 year survival rate of patients is 72.5%.^22^ Yu and Liu observed patients with SCC have a longer 5 year survival rate and a longer median survival time when submitted to comprehensive treatment (both surgery and radiotherapy) compared with patients that received a single treatment (either surgery or radiotherapy).^22^

Treatment of SNIP consists of a complete excision of tumor and removal of adjacent mucosa and mucoperiosteum at the site of tumor origin.^23^, ^24^ Microscopic foci of papilloma cells can be concealed on subjacent bone, therefore, drilling the bone at the site of origin of the tumor can contribute to reduced risk of recurrence.^5^ Success rate of endoscopic surgery for papilloma resection is described as about 95%, with less morbidity in comparison to the external approach.^23^

Recurrence rate for inverted papilloma has been described in up to 78%^3^ in the literature and some risk factors for recurrence were identified, such as tobacco exposure, size of tumor, high hyperqueratosis, squamous hyperplasia, increased number of mitosis, HPV positivity, bilaterality and tumor location.^5^, ^6^, ^7^ In most cases, recurrence occurs in the first three years after surgery, although, there are reports of recurrence after 10 years.^5^, ^6^, ^7^, ^12^ In this study, recurrence rate for inverted papilloma was 34.09% and mean time for recurrence was 24.6 months, consistent with findings from other tertiary hospitals.^24^

There is no consensus regarding the ability of Krouse staging system to estimate inverted papilloma recurrence rate. Lisan et al. found a 51% increased risk of recurrence for SNIP classified as Krouse Stage T3 disease when compared with Stage T2. In our study, 80% of recurrent lesions were classified as Stage T3, which is consistent with this finding.^25^ However, a recent study comparing different staging systems found that systems that classify SNIP based on involvement of specific paranasal sinuses have unfavorable prognostic abilities for recurrence.^11^ Meng et al. proposed a new origin site-based system to classify SNIP and have found a good correlation between SNIP stage and recurrence rate.^26^

Recurrent tumors have been described as more aggressive and with higher further recurrence rate, in comparison to primary lesions.^9^ This trend was found in this study if we observe that 66.67% of papillomas that suffered malignant transformation had undergone previous sinus surgery. We observed origin site of recurrent papillomas seems to have the same distribution as primary lesions. This might suggest recurrence can result from incomplete removal of original tumor, which was also demonstrated in previous studies.^9^, ^10^

## Conclusion

Although sinonasal papillomas are benign lesions, the high recurrence rate and malignancy potential allow us to consider them aggressive tumors. In a tertiary hospital in São Paulo the recurrence rate was 34.09% with mean time to recurrence 24.6 months, consistent with current literature. Recurrence after 10 years, as we found in this study, implies the need for long-term followup. High recurrence rate and high malignant transformation rate observed are may be due to the large tumor extension (most of them Staged T3 and T4), secondary to population’s poor access to health system, in developing countries.

## Conflicts of interest

The authors declare no conflicts of interest.
